# Dissecting Allele Architecture of Early Onset IBD Using High-Density Genotyping

**DOI:** 10.1371/journal.pone.0128074

**Published:** 2015-06-22

**Authors:** David J. Cutler, Michael E. Zwick, David T. Okou, Sampath Prahalad, Thomas Walters, Stephen L. Guthery, Marla Dubinsky, Robert Baldassano, Wallace V. Crandall, Joel Rosh, James Markowitz, Michael Stephens, Richard Kellermayer, Marian Pfefferkorn, Melvin B. Heyman, Neal LeLeiko, David Mack, Dedrick Moulton, Michael D. Kappelman, Archana Kumar, Jarod Prince, Promita Bose, Kajari Mondal, Dhanya Ramachandran, John F. Bohnsack, Anne M. Griffiths, Yael Haberman, Jonah Essers, Susan D. Thompson, Bruce Aronow, David J. Keljo, Jeffrey S. Hyams, Lee A. Denson, PRO-KIIDS Research Group, Subra Kugathasan

**Affiliations:** 1 Department of Human Genetics, Emory University, Atlanta, Georgia, United States of America; 2 Department of Pediatrics, Emory University, Atlanta, Georgia, United States of America; 3 Hospital for Sick Children, Toronto, Ontario, Canada; 4 Department of Pediatrics, University of Utah, Salt Lake City, Utah, United States of America; 5 Icahn School of Medicine, Mount Sinai Hospital, New York, New York, United States of America; 6 Children’s Hospital of Philadelphia, Philadelphia, Pennsylvania, United States of America; 7 Nationwide Children’s Hospital, Columbus, Ohio, United States of America; 8 Goryeb Children’s Hospital, Morristown, New Jersey, United States of America; 9 Cohen Children’s Medical Center, New Hyde Park, New York, United States of America; 10 Pediatric Gastroenterology, Mayo Clinic, Rochester, Minnesota, United States of America; 11 Baylor College School of Medicine, Texas Children’s Hospital, Houston, Texas, United States of America; 12 Riley Children’s Hospital, Indiannapolis, Indianapolis, United States of America; 13 University of California, San Francisco, California, United States of America; 14 Hasbro Children’s Hospital, Providence, Rhode Island, United States of America; 15 Children’s Hospital of Eastern Ontario, Ottawa, Canada; 16 Vanderbilt Children’s Hospital, Nashville, Tennessee, United States of America; 17 University of North Carolina, Chapel Hill, North Carolina, United States of America; 18 Cincinnati Children’s Hospital Medical Center, Cincinnati, Ohio, United States of America; 19 Children’s Hospital of Boston, Boston, Massachusetts, United States of America; 20 Children Hospital of Pittsburgh, Pittsburg, Pennsylvania, United States of America; 21 Connecticut Children’s Medical Center, Hartford, Connecticut, United States of America; 22 PRO-KIIDS Research Group, New York, New York, United States of America; CWRU/UH Digestive Health Institute, UNITED STATES

## Abstract

**Background:**

The inflammatory bowel diseases (IBD) are common, complex disorders in which genetic and environmental factors are believed to interact leading to chronic inflammatory responses against the gut microbiota. Earlier genetic studies performed in mostly adult population of European descent identified 163 loci affecting IBD risk, but most have relatively modest effect sizes, and altogether explain only ~20% of the genetic susceptibility. Pediatric onset represents about 25% of overall incident cases in IBD, characterized by distinct disease physiology, course and risks. The goal of this study is to compare the allelic architecture of early onset IBD with adult onset in population of European descent.

**Methods:**

We performed a fine mapping association study of early onset IBD using high-density Immunochip genotyping on 1008 pediatric-onset IBD cases (801 Crohn’s disease; 121 ulcerative colitis and 86 IBD undetermined) and 1633 healthy controls. Of the 158 SNP genotypes obtained (out of the 163 identified in adult onset), this study replicated 4% (5 SNPs out of 136) of the SNPs identified in the Crohn’s disease (CD) cases and 0.8% (1 SNP out of 128) in the ulcerative colitis (UC) cases. Replicated SNPs implicated the well known *NOD2* and *IL23R*. The point estimate for the odds ratio (ORs) for *NOD2* was above and outside the confidence intervals reported in adult onset. A polygenic liability score weakly predicted the age of onset for a larger collection of CD cases (p< 0.03, R^2^= 0.007), but not for the smaller number of UC cases.

**Conclusions:**

The allelic architecture of common susceptibility variants for early onset IBD is similar to that of adult onset. This immunochip genotyping study failed to identify additional common variants that may explain the distinct phenotype that characterize early onset IBD. A comprehensive dissection of genetic loci is necessary to further characterize the genetic architecture of early onset IBD.

## Introduction

The inflammatory bowel diseases (IBD) are complex, heritable disorders consisting of two main clinical forms: Crohn’s disease (CD) and ulcerative colitis (UC). Both forms share several phenotypic features and genetic susceptibility and are mainly characterized by chronic and relapsing gut inflammation. CD can occur throughout the gastrointestinal tract, whereas UC affects only the colon. Inflammatory bowel diseases affect some 1.5 million Americans [1], and ~20% of cases occur in children [2]. There is no doubt that IBD is heritable, although the 400% rise in the incidence of IBD over the last 50 years argues for an important role of gene-environment or gene-diet interactions in its pathogenesis [3–6]

Recent genome-wide association studies (GWAS) and a recent meta analysis identified over 163 IBD-associated loci, mostly in adult population of European descent: two thirds of which are shared by CD and UC, while the remaining are unique to either CD or UC [7]. Variants in these loci correlate well with IBD risk but have relatively modest effect sizes and account for a minority of the total disease variance (13.6% CD, 7.5% UC [7]) suggesting much of the missing heritability still awaits discovery. The loci with the largest known effects, *NOD2* and *IL23R*, both have odds ratios of approximately 1.5, with *NOD2* affecting only CD and *IL23R* affecting both CD and UC [7]. With at least 11 exceptionally highly powered GWAS studies [7–17] there was compelling evidence that *NOD2* and *IL23R* were the only loci in the genome with odds ratios approaching 1.5 for IBD.

The natural history of IBD exhibits substantial heterogeneity. Pediatric onset IBD (defined as the onset under 18 years old) is characterized by a greater disease severity, a higher tendency for disease manifestation in the colon, more disease extension, a change in disease location over time, and a positive family history for IBD [18–23]. These differing pathologies of early onset IBD could arise from differences in genetically attributable risk compared to adult onset IBD. Early onset diseases including IBD are expected to be more influenced by genetic factors than environmental exposures [24]. Two previous pediatric GWAS studies, although underpowered, identified loci that have been replicated in adult onset IBD GWAS studies [12, 13]. Likewise, many established GWAS loci ascertained in adults did not distinguish early from later onset CD [25]. Here we used the Immunochip, a high-density custom array designed for fine mapping and deep replication of ~200,000 established genome-wide significant SNPs (within 186 loci) identified by GWAS for 12 autoimmune and inflammatory diseases, to determine how the allele architecture of early onset IBD compares to that of adult onset IBD.

## Results

**Fig 1:**
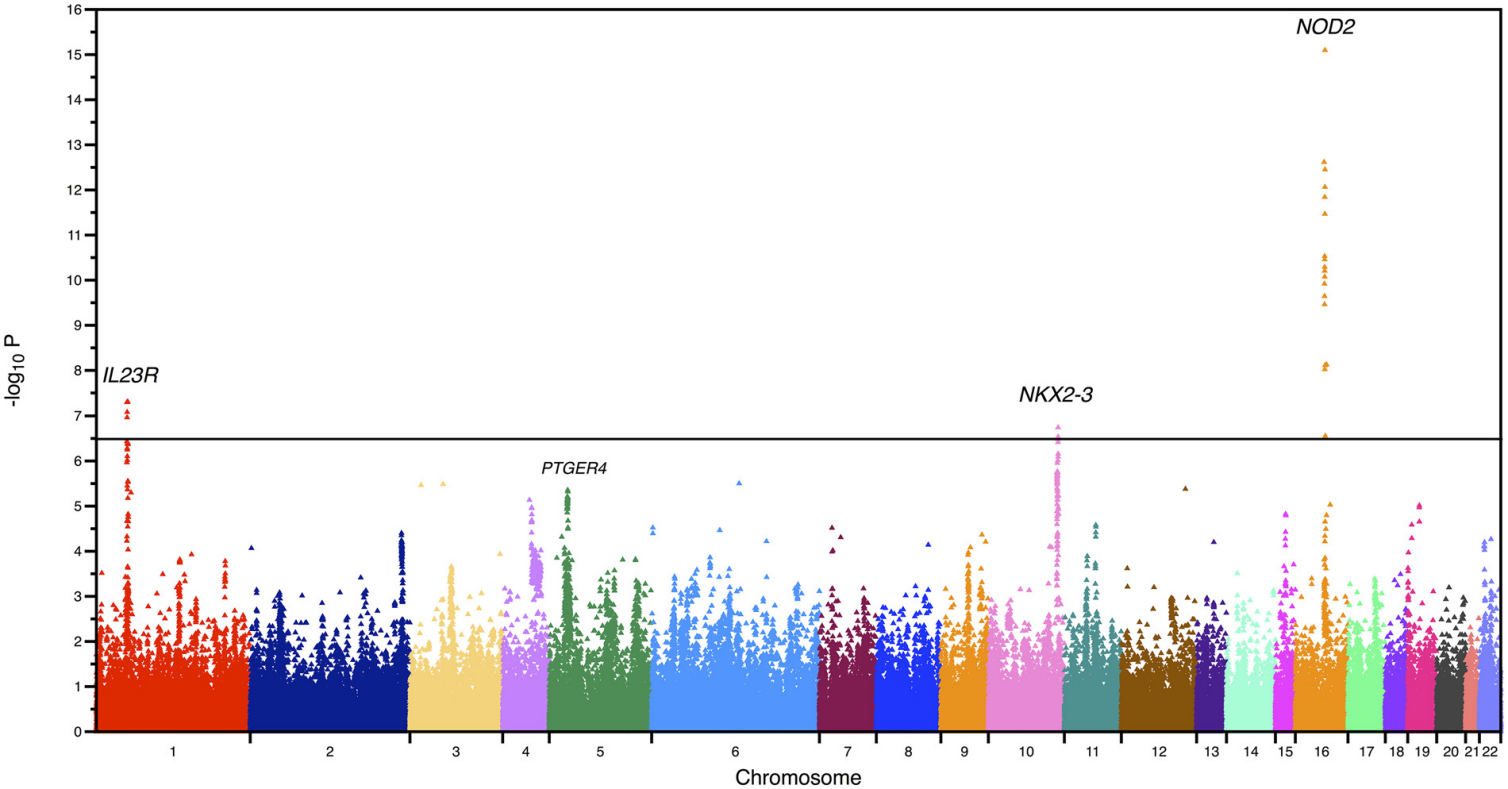
Manhattan plot of SNP association p-values result for CD vs controls within pediatric onset IBD. The horizontal black line represent the thresholds of *P* = 3.57 x 10^–7^ for Bonferroni significance

We performed a genome-wide scan of our collection of early-onset CD cases vs controls (S1 Table) and early-onset UC vs controls (S2 Table). Fig 1 shows the results of CD cases vs controls for the ~140,000 SNPs that passed QC procedures. Highly conservative Bonferroni correction for multiple testing suggests any SNP p-value below 0.05/140000 = 3.57 x 10^–7^ is experiment-wide significant, which corresponds to a-log_10_(p) of approximately 6.5. Some of the most significant loci are labeled for the CD vs controls analysis and are shown in Fig 1. None of the loci in the remaining cases (IBDminusCD) vs controls analysis exceeded our conservative experiment-wide threshold for statistical significance, which reflects the limited sample size of this portion of our study (S1 Fig).

After performing quality control, we successfully obtained genotypes for 158 of the top 163 SNPs from Jostins et al. [7] previously reported to be associated with CD and/or UC (S4 Table). For CD, 2 of 30 CD SNPs and 3 of 106 IBD SNPs successfully replicated at a Bonferroni corrected threshold of 0.0003. For UC, 0 of 22 UC SNPs and 1 or 106 IBD SNPs successfully replicated at a Bonferroni corrected threshold of 0.0003.

The limited number of Jostins et al. SNPs that replicated in our study is expected if reported effect sizes from a largely adult cohort are similar to those in our pediatric cohort. To better assess this pattern, we asked if our point estimate for the odds ratios (ORs) fell within the confidence intervals reported in the more highly powered study reported by Jostins et al. For CD, the ORs for 128 SNPs were included within their respective Jostins’ confidence intervals, while ORs for 8 SNPs fell outside the Jostins’ estimated confidence intervals (S3 Table). The point estimates for the CD ORs from our study were greater for 3 of 8 SNPs as compared to those reported in Jostins et al. Of note, our OR point estimate for *NOD2*, the most strongly associate locus in both studies, is modestly larger and outside the CIs reported by Jostins (1.9 vs 1.6). A similar pattern was observed for UC, where the ORs for 118 SNPs were included within the CIs, while the ORs for 10 SNPs were found outside the Jostins’ CIs (S4 Table). The point estimates for the UC ORs from our study were greater for only 1 of 10 SNPs as compared to those reported in Jostins et al. We next tested whether a polygenic liability score could predict the age of onset for either CD or UC. For CD, we observed a weak, but statistically significant relationship (p < 0.03, R^2^ = 0.007, S6 Fig). For UC, we did not observe a statistically significant relationship (S7 Fig).

We sought next to assess the genetic architecture differences between very early onset (VEO) cases (0 to < 10 years) as compared early onset (EO) cases (10 to < 17 years). The analysis of CD did not reveal any genome-wide significant findings (S2 Fig, S5 Table) between VEO and EO cases. Among the top findings, a SNP in the *IL-19* locus reached a nominal significance of 7 x 10^–5^. The remaining cases analysis (IBDminusCD) also failed to reveal any statistically significant findings (S3 Fig and S6 Table).

## Discussion

There is strong evidence to support the genetic contribution and heritability to the pathogenesis of IBD [26–33]. Recent efforts to understand the genetics of IBD have identified over 163 IBD-associated loci, mostly in adult populations of European descent [7]. These results explain only 13.6% of CD and 7.5% of UC total disease variance, showing that these GWAS loci contribute only little towards the explained IBD heritability. The early onset IBD population presents with a distinct disease physiology, course and risks [18–23], suggesting the possibility of a different underlying molecular mechanism [34]. To date, GWAS has failed to identify loci that can distinguish early onset from adult IBD [12, 13, 25]. To determine how the allele architecture of early onset compares to that of adult onset, we performed fine mapping of established GWAS loci in a well characterized prospectively recruited pediatric onset IBD cases and control study, using a high-density custom array (Immunochip).

Our strongest associations for CD replicate alleles found at the *NOD2* and *IL23R* loci. This study suggests that common risk variants at both *NOD2* and *IL23R* loci act in a similar fashion in both early and later onset populations. This also confirms previous reported associations of *NOD2* and *IL23R* with both pediatric and adult onset CD [35–37]. Of interest, our OR point estimate for *NOD2*, the most strongly associate locus in both studies, is modestly larger and slightly outside the CIs reported by Jostins (1.9 vs 1.6). IBD is a complex disease whose heritability is influenced by only a small number of loci with large effect sizes (in this case *NOD2* and *IL23R*), but a relatively large number of loci whose effects are detectable in very large GWAS. This scenario has in fact been reported for common trait under biotic selection [38].

Only few in this study reached genome-wide significance threshold. Most of the loci previously reported by Jostins et al. did not replicate in our smaller pediatric cohort. For CD, we observed a weak, but statistically significant relationship between a polygenic liability score and age of onset (p < 0.03, R^2^ = 0.007, S6 Fig). For UC, we did not observe a statistically significant relationship (S7 Fig). At the vast majority of loci, our estimated effect sizes fell within the confidence intervals reported in adult onset [7]. Over 91% (128 SNPs) of the SNPs that explain slightly less than 20% of the CD heritability in the adult onset had similar estimated effect sizes in early onset CD cases. Similarly, close to 89% (118 SNPs) of those UC SNPs had similar estimated effect sizes in early onset UC cases.

While our sample size was significantly smaller than that used by Jostins et al, this finding suggests an overall similar genetic architecture between adult and pediatric onset CD, IBD, and UC. Consistent estimates of SNP effect sizes in separate adult and early onset IBD cohorts suggests that there are likely to be great similarities in the overall pathogenesis and underlying molecular pathways of IBD. Furthermore, if early-onset susceptibility alleles exist, they will be difficult to discover in the absence of extremely large studies. As most common loci ascertained by GWAS, the loci in this study have small effect size, which are known to additively explain only a small proportion of the heritability of complex diseases [39]. One explanation for the “hidden heritability” is that additional or true causal variants could be present within known loci but they may be poorly tagged and cannot be revealed by the GWAS SNPs surveyed [40]. If true, this suggests that some of the reported clinical heterogeneity within IBD and differences in phenotypic characteristics between early and late onset could be driven by low frequency to rare variants, which will only be discovered through whole-exome sequencing, whole-genome sequencing, or family-based studies [41].

Pediatric onset IBD can be further separated into 2 distinct sub-groups based on the Paris classification; very early onset (VEO) 0 to < 10 years and early onset (EO) 10 to < 17 years [42]. Very early onset (VEO) IBD is characterized by a greater disease severity, a higher tendency for disease manifestation in the colon, more disease extension, a change in disease location over time, and a positive family history for IBD [18–23]. Our assessment of the genetic architecture differences between VEO and EO IBD cases revealed interesting differences in the allele architecture but no genome-wide significant findings (Figs S2, S3 and S5 and S6 Tables), which may reflect the limited analysis power for this sub-groups. One SNP in the *IL-19* locus reached a nominal significance for VEO CD analysis. IL-19 belongs to IL-10 superfamily and is known to suppress bacteria induced macrophage pro-inflammatory cytokine production in the host following injury to intestinal epithelial cells [43]. IL10 is an established genetic risk for IBD, including a role of IL10 receptor variants in VEO and EO IBD [44, 45]. It is not clear if the nominal significant IL19 signal is independent or exhibited via IL10 as both loci are linked together through genetic similarity and intron-exon gene structure. Owing to the limitation of GWAS and the expectation of higher genetic load and reduced environmental modifiers in VEO cases [24, 46], the allelic architecture of VEO must be characterized by low to rare frequency variants with greater penetrance not detectable by GWAS, or are simply located in portions of the genome not previously associated with IBD, and therefore not represented on the Immunochip. Rare variants are predicted to vastly outnumber common variants in the human genome [47].

This study showed that the allelic architecture of common susceptibility variants seen in adult onset is also shared with early onset cases, suggesting similarities in the overall pathogenesis of IBD. GWAS have limited utility in identifying low frequency to rare variants and true causal genetic variants associated with distinct phenotypes within the inflammatory bowel complex disease. It is therefore necessary that a more comprehensive dissection of the human genome or known loci be done to further understand the genetic architecture of early onset IBD. Sequencing-based methods are better at systematically ascertaining both common and rare SNPs.

## Materials and Methods

### Samples

We present a multi-center collaborative study of pediatric inflammatory bowel disease (the RISK cohort). IBD subjects were obtained from the RISK Study. The RISK Study is an ongoing, prospective observational inception IBD cohort funded by the Crohn's and Colitis Foundation of America (CCFA) and currently includes 28 pediatric gastroenterology centers in North America. Children and adolescents younger than 17 years newly diagnosed with inflammatory bowel disease (IBD) were eligible for enrollment in RISK between November 2008 and June 2012. All subjects presented are of European ancestry.

All patients were required to undergo baseline colonoscopy and confirmation of characteristic chronic active colitis/ileitis by histology prior to diagnosis and treatment, with the recording of findings in standardized fashion. Once standard and published guidelines were met, patients were diagnosed with CD and UC and inflammatory bowel disease-undetermined (IBD-U). A firm and consistent diagnosis of IBD was required during the one-year follow-up for inclusion into this study. At enrollment and during ongoing prospective follow-up, clinical and laboratory data were obtained for each enrolled patient and submitted to a centralized data management center. All patients were managed according to the dictates of their physicians, not by standardized protocols.

The study populations were derived from three sources, including 1) 1401 cases with IBD (after QC and removal of non-Europeans: CD = 801; UC = 128; IBD-U = 86) recruited from the RISK cohort, (2) 1,663 healthy controls consisting of 831 pediatric healthy children recruited from Cincinnati Children's Hospital Medical Center (CCHMC), 647 healthy adults from Utah, and 185 non-IBD subjects from the RISK Study. Control subjects did not have any chronic autoimmune or inflammatory disorders. Most of the control subjects were part of other GWAS and Immunochip studies [48]. The institutional review board at Emory University approved the protocol. Written informed consent was provided by all parents/caregivers, and written assent was obtained from children as appropriate.

### High-density Genotyping with the Illumina Immunochip

The Immunochip, a custom Illumina Infinium High-Density array, contains 196,524 polymorphisms (718 small insertion/deletions, 195,806 SNPs). It was initiated by the Welcome Trust Case-Control Consortium and designed for deep replication of established autoimmune and inflammatory disease loci identified by GWAS of common variants using data from the 1000 Genomes Project and any other available disease-specific resequencing data. The Immunochip Consortium selected 186 distinct loci containing markers reaching genome-wide significance (P < 5 × 10^−8^) from 12 diseases (autoimmune thyroid disease, ankylosing spondylitis, Crohn's disease, celiac disease, IgA deficiency, multiple sclerosis, primary biliary cirrhosis, psoriasis, rheumatoid arthritis, systemic lupus erythematosus, type 1 diabetes, and ulcerative colitis). For each disease, ~3,000 SNPs were selected from available GWAS data for deep replication, as well as to cover strong candidate genes. Samples were genotyped using the Immunochip according to Illumina's protocols at laboratories at Emory University and The Feinstein Institute for Medical Research and Cincinnati Children's Hospital Medical Center (Utah samples and Cincinnati controls).

### Genotype Determination, Data Cleaning, and Statistical Analyses

All chip images were merged into a single batch for simultaneous genotype calling with BeadStudio. Included in these samples were 133 replicates (the same sample run twice), and 33 parent-offspring pairs. These replicates and family members were used to directly test for genotyping error [49]. Samples were tested for cryptic (unexpected) relatedness, incorrect genders, overall data completeness, and overall heterozygosity; samples were excluded if they had less than 90% data completeness, differed by more than three standard deviations from the mean heterozygosity for the study, had the wrong gender, or were unexpectedly the first-degree relative of any other sample in the study. Approximately 15% of our samples were dropped for one or more of these reasons.

BeadStudio reported genotypes for 189,012 autosomal SNPs. Any SNP with more than 2% missing data, only one allele present (i.e. not segregating), any detectable genotype error (either through duplicates or parent-offspring), or a Hardy-Weinberg p-value less than 10^–5^ in controls was dropped. This resulted in a final dataset of approximately 140,000 autosomal SNPs. Over 40% of the dropped SNPs were dropped simply because there was only one allele present (~20,600 SNPs).

For this study, we performed three main association analyses. We first evaluated patients with a diagnosis of CD with matched controls. We then evaluated all remaining cases (IBDminusCD), which consisted of cases with UC or other indeterminate IBD diagnosis (but not CD). Finally, we performed association analyses using very early onset (VEO) CD or UC /IBD-U labeled as “cases” contrasted with matched early onset (EO) CD or UC / IBD-U labeled as “controls”. Matching of cases and controls was done by determining principal components (PC) with Eigenstrat [50, 51], plotting PC1 against PC2, followed by visual inspection and elimination of outlier samples. Four successive rounds were necessary until a satisfactory matching of CD cases/controls (S4 Fig) and UC (IBDminusCD) cases/controls (S5 Fig) was obtained.

After all SNP and sample removal, 1,633 controls, 801 CD, and 207 UC / IBD-U individuals remained. From the 801 CD cases, we performed a second association with 267 VEO CD “cases” contrasted with 525 EO CD “controls”. From the 207 UC cases, there were 62 VEO UC / IBD-U “cases” contrasted with 143 EO UC / IBD-U “controls”. We excluded 7 CD and 2 UC samples because of indeterminate age of diagnosis. All samples were unrelated to one another. All association analysis was performed with PLINK 1.0.7 via a logistic regression [52], additive model, adjusting for the first five principal components of ancestry as determined by Eigenstrat. Replication of the top SNPs from Jostins et al. successfully genotyped in this study was assessed by performing a Bonferroni correction for 158 tests (0.05/158), resulting in a threshold of 0.0003. The complete summary for all SNP associations for CD (S1 Table) and UC (S2 Table) are contained in the supplemental materials. The summary for the 158 top SNPs for CD (S3 Table) and UC (S4 Table) are also included in the supplemental materials.

Polygenic liability scores were calculated assuming an underlying normal distribution of liability with disease (CD or UC) state representing a threshold on the continuous liability scale [53]. To do so, we first assumed the odds ratio estimated by Jostins’ et al [7] is the true odds ratio for the identified allele. Using the observed allele frequency in controls from this study, and an assumed prevalence for CD of 5 in 10,000, and for UC of 1 in 10,000, independent of sex, the additive effect on liability of each of the (163) Jostins’ identified SNPs was calculated [53]. Final polygenic liability for each sample was calculated by summing the additive affects for both alleles at all 163 loci. Thus we assume both additive dominance and additive epistasis on the liability scale. This polygenic liability score was regressed against age of onset for all CD and UC cases separately. While our cohort is purely pediatric onset (less than 18 years) only about 10% of the Jostins et al cohort were under 18 years of age at onset (personal communication with Dr Judy Cho).

The data of this study have been deposited into the Odum Institue Dataverse Network hosted at UNC (http://arc.irss.unc.edu/dvn/) and is accessible through the accession number doi:10.15139/S3/11991.

## Supporting Information

S1 Fig Manhattan plot of SNP association p-values result for IBD-minusCD vs Controls within pediatric onset IBD. The horizontal black line represent the thresholds of *P* = 3.57 x 10^–7^ for Bonferroni significance.
(TIFF)

S2 Fig Manhattan plot of the association analysis for CD between VEO taken as cases and EO taken as “control”. The horizontal black line represent the thresholds of *P* = 3.57 x 10^–7^ for Bonferroni significance.
(TIFF)

S3 Fig Manhattan plot of the association analysis for IBD-minusCD between VEO taken as cases and EO taken as “control”. The horizontal black line represent the thresholds of *P* = 3.57 x 10^–7^ for Bonferroni significance.
(TIFF)

S4 Fig Principal component analysis (PCA) plot of the pediatric CD and control group. Yellow and blue dots represent CD and control respectively.
(TIFF)

S5 Fig Principal component analysis (PCA) plot of the pediatric IBD-minusCD and control group. Yellow and blue dots represent IBD-minusCD and control respectively.
(TIFF)

S6 Fig Relationship Between Polygenic Liability Score and Age of Onset of CD. (TIFF)

S7 Fig Relationship Between Polygenic Liability Score and Age of Onset of UC. (TIFF)

S1 Table Summary of SNP association findings for CD analysis. (CSV)

S2 Table Summary of SNP association findings for UC analysis. (CSV)

S3 Table Summary of CD association findings from this study for top SNPs reported in Jostins et al. 2012 (XLSX)

S4 Table Summary of UC association findings from this study for top SNPs reported in Jostins et al. 2012 (XLSX)

S5 Table Summary of association findings from this study for CD comparing very early onset (VEO) with early onset (EO) patients. (CSV)

S6 Table Summary of association findings from this study for UC comparing very early onset (VEO) with early onset (EO) patients. (CSV)
