# Supplementary figures and images for: Dissecting Allele Architecture of Early Onset IBD Using High-Density Genotyping
Source: PLoS One. 2015 Jun 22;10(6):e0128074. doi: 10.1371/journal.pone.0128074 (PMC4476779; doi:10.1371/journal.pone.0128074)

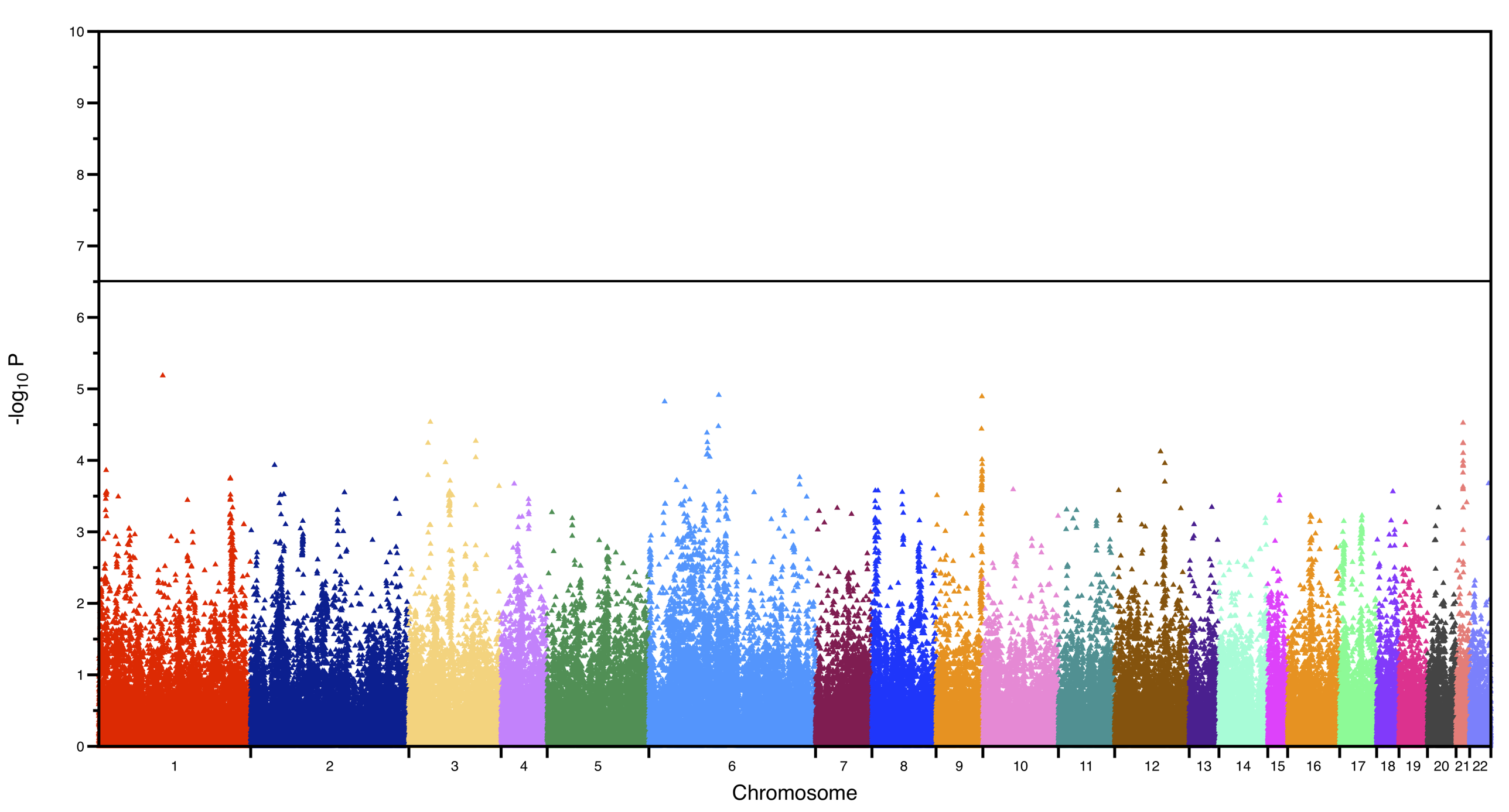

Supplement: S1 Fig — The horizontal black line represent the thresholds of P = 3.57 x 10^–7^ for Bonferroni significance. (TIFF) [file pone.0128074.s001.tiff]

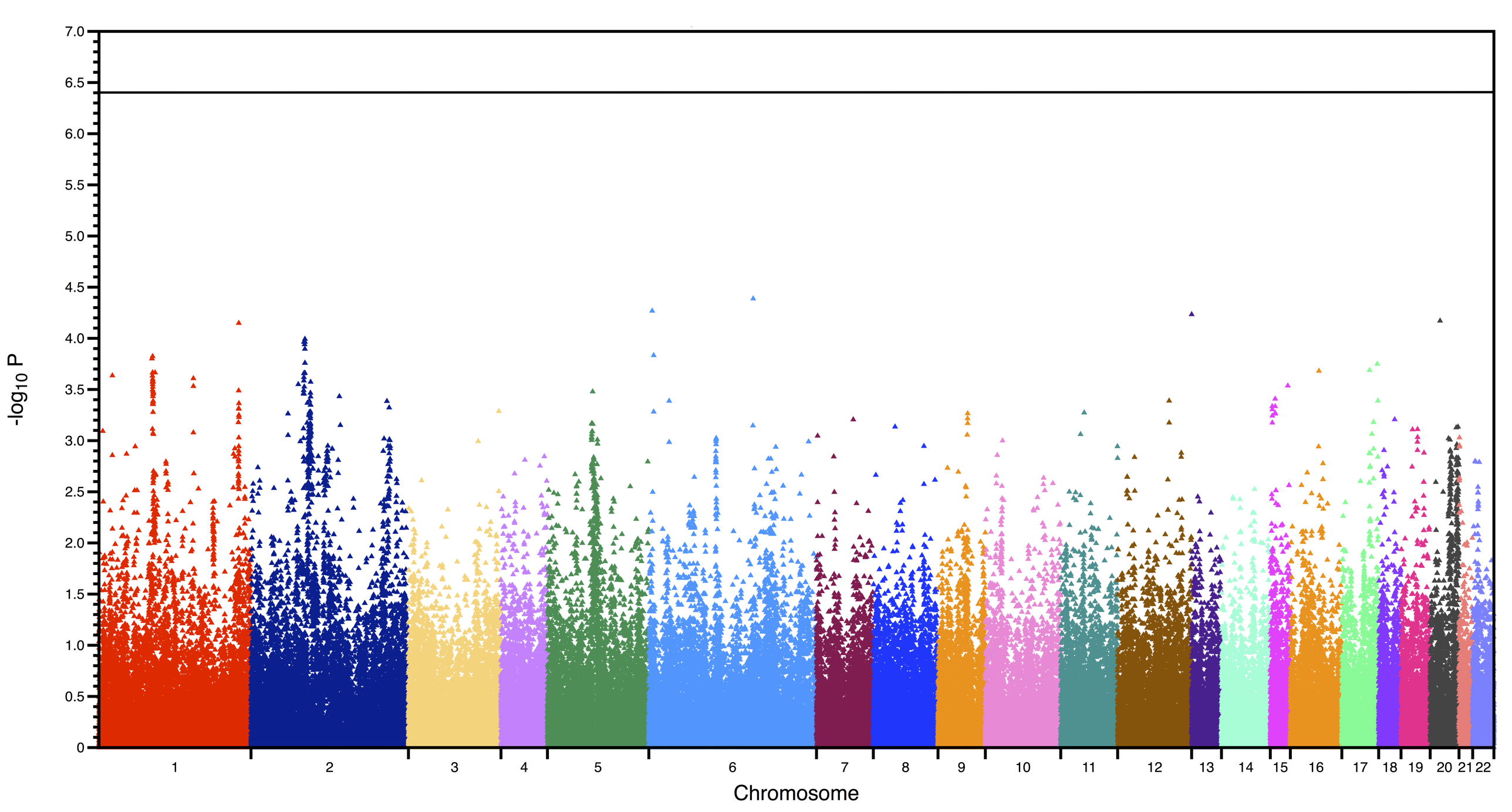

Supplement: S2 Fig — The horizontal black line represent the thresholds of P = 3.57 x 10^–7^ for Bonferroni significance. (TIFF) [file pone.0128074.s002.tiff]

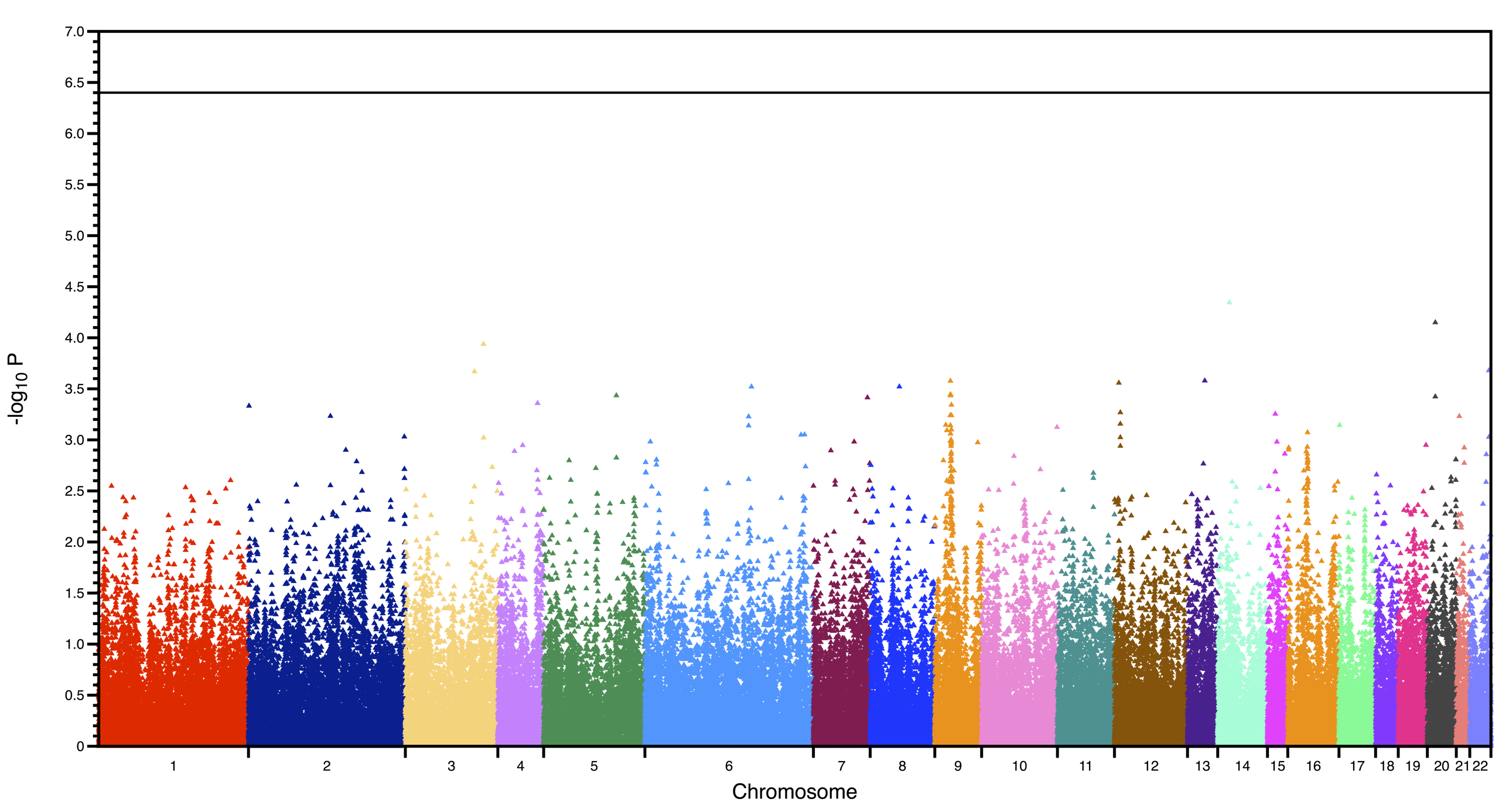

Supplement: S3 Fig — The horizontal black line represent the thresholds of P = 3.57 x 10^–7^ for Bonferroni significance. (TIFF) [file pone.0128074.s003.tiff]

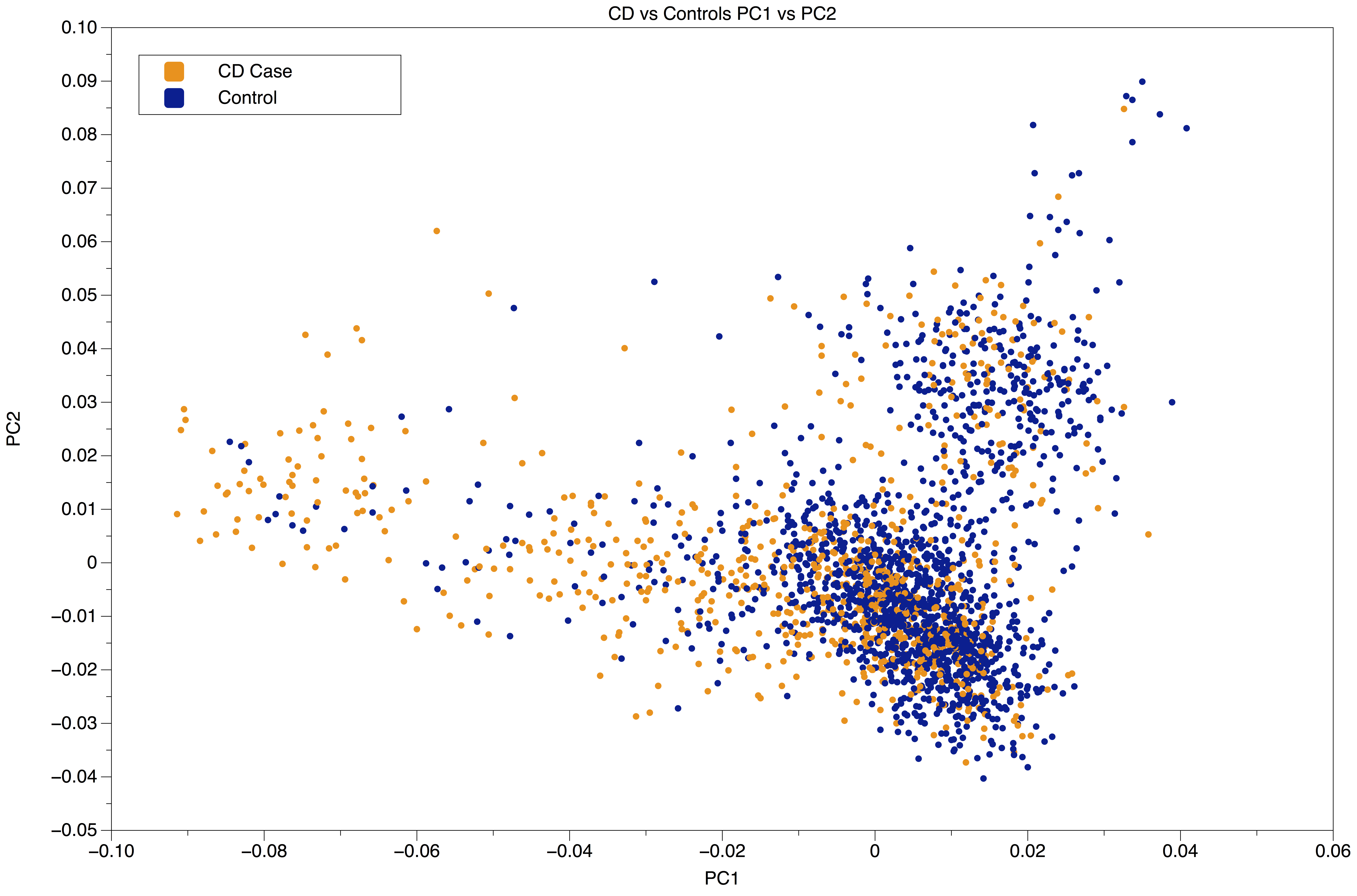

Supplement: S4 Fig — Yellow and blue dots represent CD and control respectively. (TIFF) [file pone.0128074.s004.tiff]

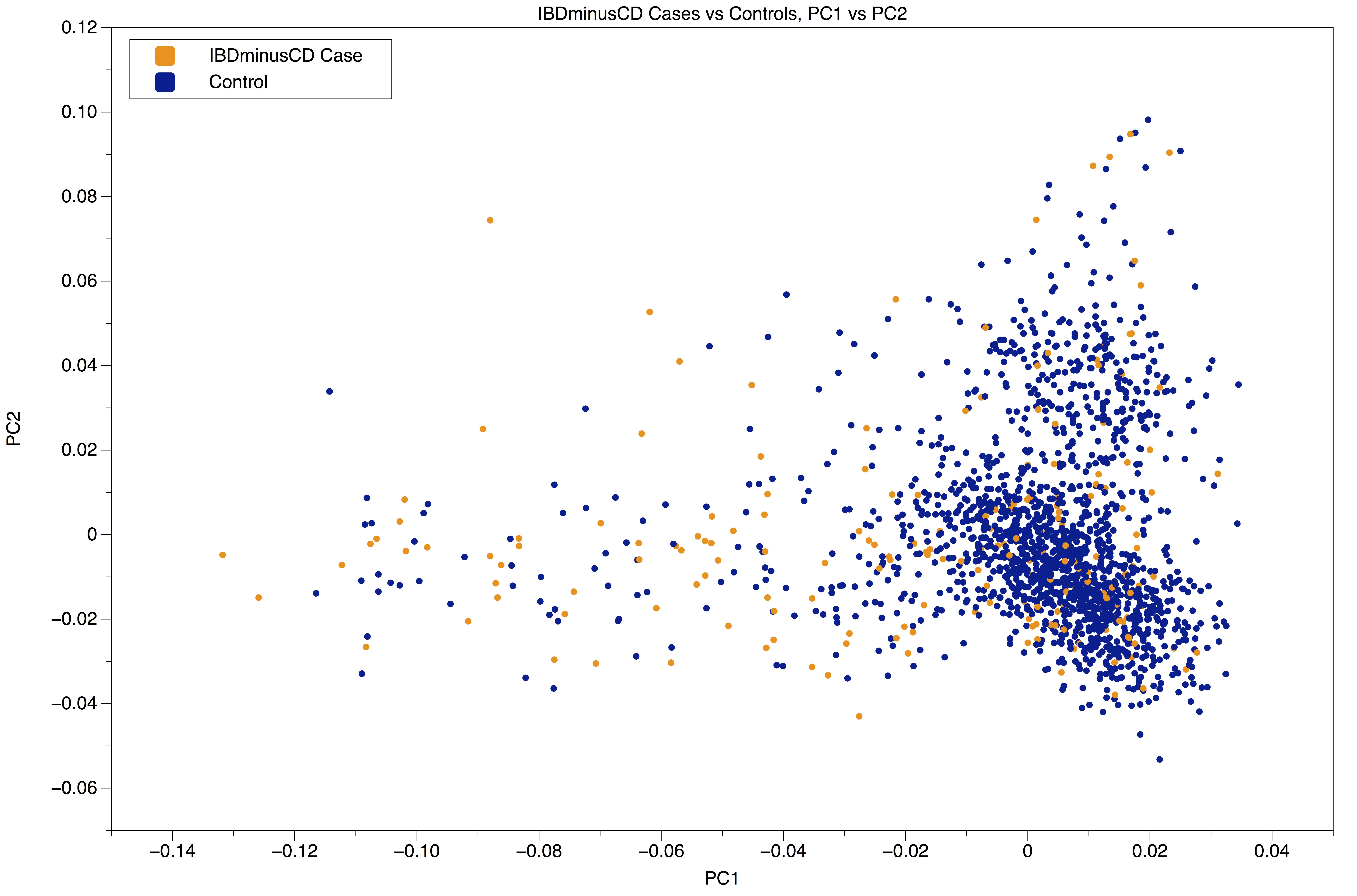

Supplement: S5 Fig — Yellow and blue dots represent IBD-minusCD and control respectively. (TIFF) [file pone.0128074.s005.tiff]

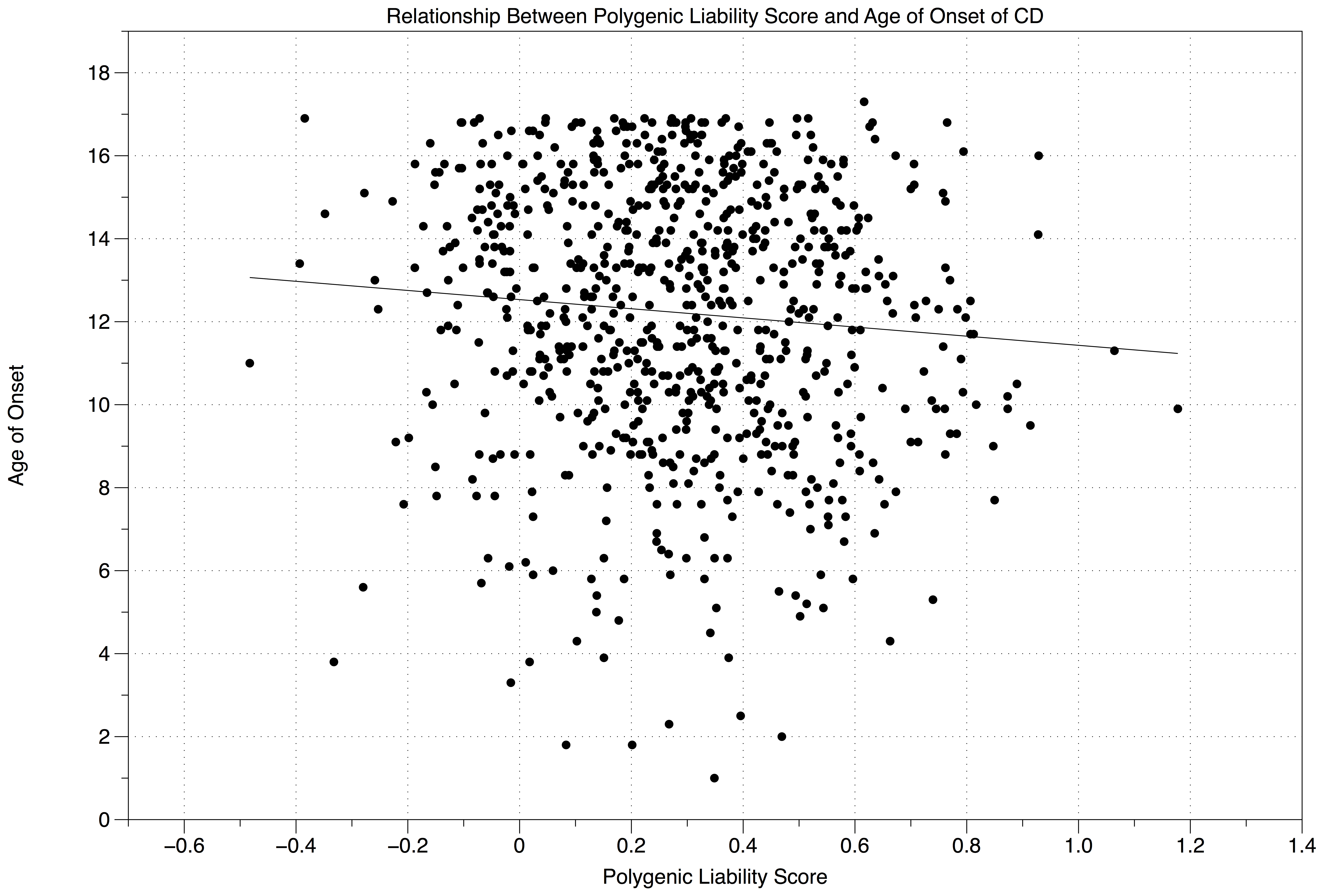

Supplement: S6 Fig — (TIFF) [file pone.0128074.s006.tiff]

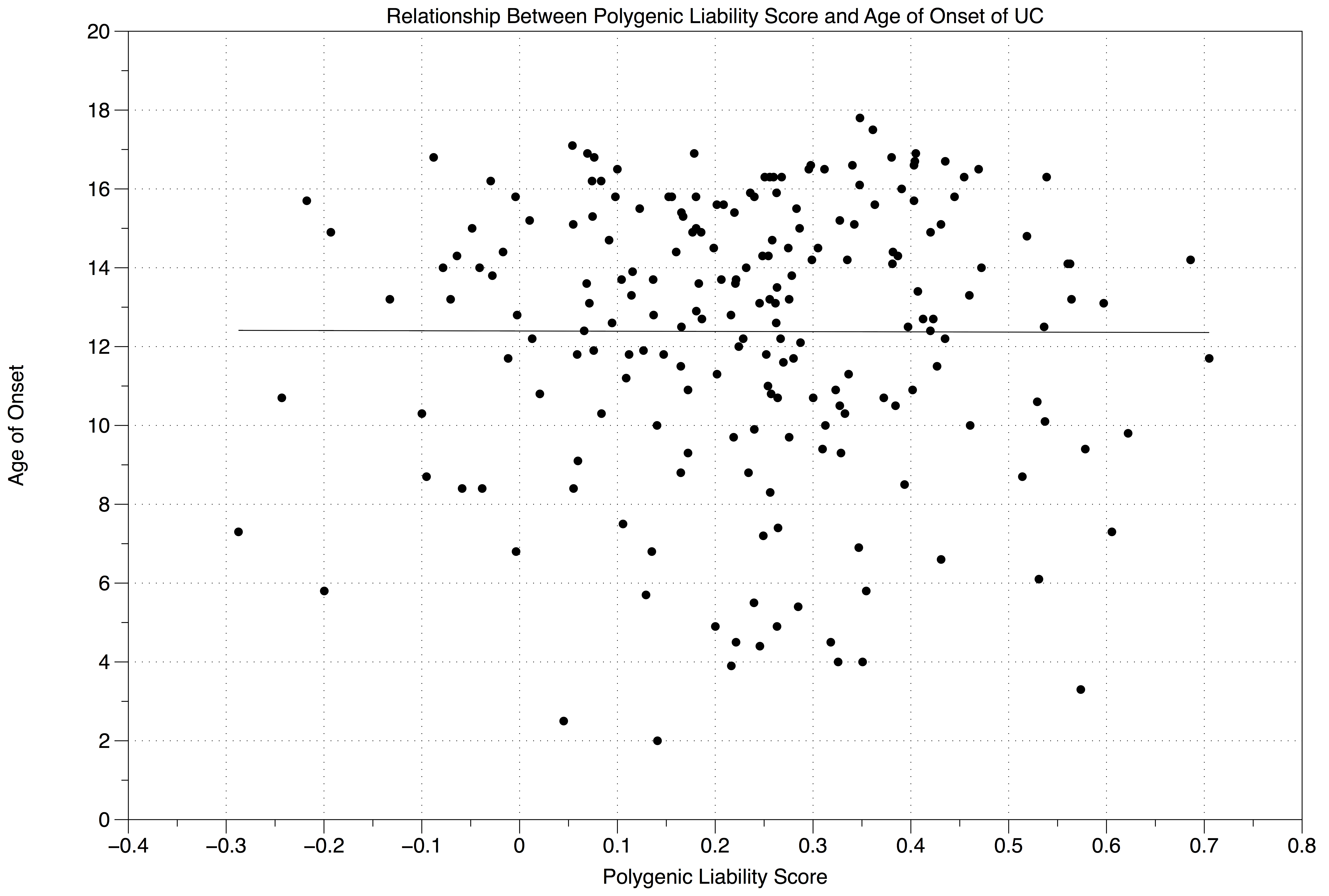

Supplement: S7 Fig — (TIFF) [file pone.0128074.s007.tiff]
